# Severe Anti-*N*-Methyl-D-Aspartate Receptor Encephalitis Under Immunosuppression After Liver Transplantation

**DOI:** 10.3389/fneur.2019.00987

**Published:** 2019-09-25

**Authors:** Franz Felix Konen, Philipp Schwenkenbecher, Konstantin Fritz Jendretzky, Martin Werner Hümmert, Florian Wegner, Martin Stangel, Kurt-Wolfram Sühs, Thomas Skripuletz

**Affiliations:** Department of Neurology, Hannover Medical School, Hanover, Germany

**Keywords:** Epstein-Barr virus, anti-NMDA receptor encephalitis, immunosuppression, liver-transplantation, autoimmune encephalitis

## Abstract

Anti-NMDA receptor encephalitis is a rare and often therapy-responsive autoimmune disease that usually affects young adults and causes neuropsychiatric symptoms. Here, we describe a 69-year-old patient who developed anti-NMDA receptor encephalitis while being under adequate immunosuppressive therapy following liver transplantation. Although a broad spectrum of different immunotherapies was applied and anti-NMDA receptor antibody titers gradually decreased, the clinical course could not be affected positively. Autoimmune encephalitis after transplantation is only described in a few cases and not well-recognized. Our case adds further evidence for anti-NMDA receptor encephalitis as the cause of neuropsychiatric symptoms even under immunosuppressive therapy in a post-transplant setting.

## Background

The anti-*N*-methyl-D-aspartate (NMDA) receptor encephalitis was first described in female patients under 50 years who developed neuropsychiatric symptoms after a non-obligate prodromal phase with flu-like symptoms (1–3). The core symptoms of the disease are cognitive deficiency, behavioral changes, dyskinesia, and seizures (2). Some patients may develop autonomic dysfunctions and reduced consciousness leading to the need of intensive care (2). This spectrum of encephalitis is associated with antibodies that target extracellular epitopes of cell-surface or synaptic proteins such as the GluN1 subunit of the NMDA receptor (2). Associated neurological syndromes often respond to immunotherapy, achieving substantial, or complete recovery in >75% of the patients (2).

Since a strict immunosuppressive treatment after solid organ transplantation is mandatory, it could be assumed that those patients have only marginal risk in developing autoimmune encephalitis. However, anti-NMDA receptor encephalitis was described in five post-transplant cases (4–8). Here, we report the first case of anti-NMDA receptor encephalitis after liver transplantation during sufficient anti-rejection immunosuppressive therapy.

## Case Presentation

A 69-year-old woman presented with a 10-day history of progressive mental impairment to the emergency department of a community hospital. Acquaintances of her had observed that she withdraw from social life, barely spoke, and appeared mentally absent. The clinical examination showed that she was aphasic and not oriented. The remaining neurological examination was unremarkable.

A decade before onset of the neurological symptoms, she was diagnosed with liver cirrhosis due to chronic hepatitis c virus infection of which she suffered for 13 years and received an allogenic transplant. The cause of hepatitis c virus infection remained unclear. From then on, she was permanently on immunosuppressive therapy with tacrolimus and mycophenolate mofetil. At the time of onset of neurological symptoms, tacrolimus was administered with 1.5 mg/day and mycophenolate mofetil with 1,000 mg/day. Blood dosage of tacrolimus was 2.2 μg/L, and blood dosage of mycophenolate mofetil was not examined. White blood cell count and distribution revealed normal values for leukocytes (8,300/μl) and lymphocytes (1,300/μl).

**Figure 1:**
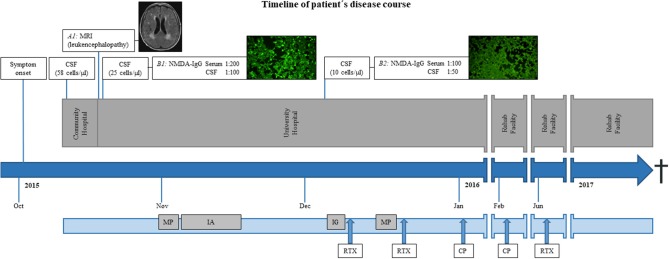
Timeline of patient's disease course. *(A1)* Exemplary axial fluid-attenuated inversion recovery (FLAIR) magnetic resonance imaging (MRI) of the brain demonstrated leukoencephalopathy but no signs of inflammation. Immune-fluorescence microscopy of anti-NMDA receptor staining with high (*B1*) and low titers (*B2*) in serum (depicted) and CSF. Bright green cells represent an antibody-antigen-interaction (*B1*) while dim cells do not reveal such interaction (*B2*). CSF, cerebrospinal fluid; MRI, magnetic resonance imaging; MP, methylprednisolone; IA, immunoadsorption-therapy; IG, intravenous immunoglobulins; RTX, rituximab; CP, cyclophosphamide; Anti-NMDA receptor, Anti-N-methyl-D-aspartate receptor.

After first admission, contrast enhanced magnetic resonance imaging (MRI) of the brain demonstrated leukoencephalopathy but no signs of a brain tumor or encephalitis. Basic cerebrospinal fluid (CSF) diagnostic revealed pleocytosis (58 cells/μl) and an elevated protein level (695 mg/L), while the lactate concentration was within the normal range (2.1 mmol/L). A viral encephalitis was assumed and the patient was treated intravenously with acyclovir. In the course of disease, she suffered from generalized epileptic seizures and an anticonvulsive therapy with levetiracetam 2 × 500 mg/day was initiated. The patient was then transferred to our university hospital. Follow-up MRI showed no change (Figure 1, *A1*) and electroencephalogram (EEG) was unremarkable. Due to a rapid progressive disturbance of consciousness within a week that led to a vegetative state, she had to be treated on the intensive care unit. The immunosuppressive therapy was changed from tacrolimus and mycophenolate mofetil to intravenous hydrocortisone. One month after immunosuppression with intravenous hydrocortisone, blood dosage of tacrolimus was 2.5 μg/L and that of mycophenolate mofetil was 3.2 mg/L, while the according white blood cell count and distribution revealed values within the reference range for leukocytes (6,600/μl) and lymphocytes (1,200/μl). Since C-reactive protein (CRP) and leucocytes were increasing, the anti-infectious therapy was changed to ganciclovir and piperacillin/tazobactam was added. CSF analysis was repeated 1 day after admission in our clinic and revealed pleocytosis (25 cells/μl, thereof 80% lymphocytes, 15% monocytes, 4% granulocytes, and 1% plasma cells), a disturbed blood–CSF barrier function (Qalbumin 12.3; protein 791 mg/L), and a lactate concentration of 2.6 mmol/L. Oligoclonal bands (OCB) restricted to CSF were found. Laboratory tests for autoimmune causes such as connective tissue diseases (antinuclear antibodies, anti-DNA antibodies, and antiphospholipid antibodies) were unremarkable. A broad diagnostic screening for infectious agents was performed. Analysis for bacterial (conventional cultural growth, mycobacterial cultures, *Treponema pallidum*, and *Borrelia burgdorferi* antibody tests), viral [antibody-specific index (AI) for herpes-simplex virus, varicella zoster virus, Epstein-Barr virus (EBV), measles virus, and rubella virus; polymerase-chain-reaction (PCR) for DNA of herpes-simplex virus, varicella zoster virus, Epstein–Barr virus, entero-virus, parecho-virus, adeno-virus, JC-virus, and human herpesvirus-6], and fungal (cultural growth and antigen test to Aspergillus and *Cryptococcus neoformans*) pathogens at different time points revealed only signs of CNS infection with Epstein–Barr virus. Polymerase-chain-reaction (PCR) analysis detected Epstein–Barr virus DNA in CSF (<3,200 copies/ml) and an elevated antibody-specific index for Epstein–Barr virus (45.3) suggesting intrathecal synthesis of EBV-specific IgG. In addition, anti-NMDAR-IgG antibodies in serum (titer 1:200) and CSF (titer 1:100) were found (Figure 1, *B1*) by using the commercially available cell-based assay of Euroimmune, confirming the diagnosis of anti-NMDAR encephalitis.

Additionally, flow cytometry of the CSF was performed to exclude post-transplant lymphoproliferative disorders (9, 10).

Corticoid treatment with 1 g of intravenous methylprednisolone was administered for 5 days followed by five courses of immunoadsorption therapy.

The patient's symptoms did not improve and thus a therapy with two cycles of intravenous immunoglobulins (60 g in total) was performed followed by a second course of methylprednisolone (1 g daily for 5 days) and two applications of rituximab (2 × 1,000 mg within 14 days). Because of the devastating disease course without any improvement, an additional immunosuppressive therapy with cyclophosphamide (750 mg/m^2^) was performed. White blood cell population count after extended immunosuppressive therapy revealed a decrease of leukocytes (2,400/μl) and lymphocytes (700/μl). As the patient experienced further epileptic seizures, the anticonvulsive treatment was expanded with valproate and lacosamid. Due to persisting epileptic seizures, lacosamid was changed to phenytoin.

Meanwhile, the gynecologic diagnostic including ovarian ultrasound remained unremarkable. Whole-body PET-CT screening showed no signs of a paraneoplastic etiology of the autoimmune encephalitis. Nevertheless, the patient underwent oophorectomy of both sides, as a rescue option that can be considered in imaging-negative anti-NMDA receptor encephalitis patients without obvious ovarian teratoma (11). Histological examination of the ovarian tissue did not detect a teratoma.

In the course of the disease, the patient slightly regained consciousness. Follow-up CSF diagnostic 8 weeks after first symptoms and 5 weeks after the first dose of steroids showed decreasing pleocytosis (10 cells/μl, thereof 90% lymphocytes and 10% monocytes) and reduced anti-NMDAR-IgG antibodies titers (1:100 in serum, 1:50 in CSF, Figure 1, *B2*). The immunosuppressive therapy was switched back to oral treatment with tacrolimus and mycophenolate mofetil and the patient was transferred to a rehab facility. The patient regained consciousness and orientation but showed a reduced general condition with cachexia and was not able to walk.

After 6 weeks, she was readmitted to our hospital for another course of cyclophosphamide and after 6 months for rituximab treatment. In the course, repeated tumor screening including cerebral, abdominal, and thoracic imaging showed no evidence of concomitant malignant diseases. However, the patient did not fully recover and died 2 years after disease onset due to septicemia (see timeline figure for overview).

## Discussion

Here, we present the first case of anti-NMDA receptor encephalitis developing despite immunosuppressive therapy after liver transplantation. Mycophenolate mofetil and tacrolimus are both highly effective drugs and were developed to prevent autoimmunity in patients after transplantation of solid organs (12–14). Mycophenolate mofetil has inhibitory effects on B- and T-cells, while tacrolimus reduces activation of T-cells (14, 15). Since the pathomechanisms of anti-NMDA receptor encephalitis are considered to be driven by complement-independent antibody effects, it could be assumed that this autoimmune disease should not occur under adequate immunosuppressive therapy with mycophenolate mofetil and tacrolimus (7). However, similar cases have been described in three patients after kidney transplantation (4, 5, 8), in one patient after repeated stem-cell transplantations in childhood and kidney transplantation in the course (7), and in one patient after heart transplantation (6). In all five published cases, immunosuppressive therapy at the time of encephalitis onset consisted of mycophenolate mofetil in addition to either tacrolimus or prednisolone (4–8). Furthermore, several reports showed that patients after allogeneic or autologous stem cell transplantation developed autoimmune diseases such as polymyositis, myasthenia gravis, Guillain–Barré syndrome, and anti-LGI1 and anti-GABAAR encephalitis, concluding that the inhibitory effect of mycophenolate mofetil on B- and T-cells and tacrolimus on T-cells might not be sufficient to prevent additional neuroimmunological diseases (16–18).

In all reported cases of anti-NMDAR encephalitis, symptoms began at least 6 years after transplantation (4–8). The symptom onset in our patient occurred even 10 years after liver transplantation. Dysfunctional immune tolerance and autoimmune phenomena are described as long-term effects of immunosuppressive therapy (16–18). It can be hypothesized that after several years of immunosuppression therapy, immune cell populations might be imbalanced, causing the breakdown of immune tolerance, in particular on the side of B-cells since chronic immunosuppression is rather T-cell targeting (12, 14).

Another interesting observation in our case is the concomitant presentation of Epstein–Barr virus DNA in CSF. In four of the five published cases, Epstein–Barr virus DNA was also found in the CSF of patients with autoimmune encephalitis (4–8). The authors suggested either reactivation of latent virus infection or first infection under immunosuppressive therapy (5, 7). An involvement of Epstein–Barr virus in autoimmune diseases such as multiple sclerosis is the subject of ongoing discussion. Casiraghi et al. propose that Epstein–Barr virus infection of brain endothelial cells could cause an upregulation of inflammatory mediators, which then induces a local breach in the brain–blood barrier and attraction of autoreactive lymphocytes into the brain (19). In analogy to this hypothesis, a similar pathomechanism with diffusion of peripheral autoreactive lymphocytes across a dysfunctional blood–brain barrier and subsequent intrathecal production of anti-NMDAR-IgG antibodies could be assumed (19).

On the other side, in another infectious disease, the herpes-simplex virus encephalitis, the herpes-simplex virus is regarded to trigger processes of autoimmunity in the CNS directly, which was demonstrated in a study involving patients with herpes-simplex encephalitis who subsequently developed autoimmune encephalitis (20, 21).

There are several other hypotheses about a possible relationship between virus infection and autoimmune encephalitis. As it is known, that EBV acquires its definite envelope by budding through the plasma membrane of infected host cells, the virus might incorporate specific components of their membrane like expressed receptors in its envelope (22). Possible targets for infection with Epstein–Barr virus are B- and T-lymphocytes, NK-cells, and epithelial cells of the nasopharyngeal and gastrointestinal area (23). If the infected host cells express the specific NMDA receptor on their surfaces as it is shown for different neuronal and extra-neuronal cells, for example, of the gastrointestinal tract, the envelope of the Epstein–Barr virus might include this receptor (24). The specific immune response against Epstein–Barr virus might also lead to an immune reaction against the virus envelope-associated NMDA receptor.

Furthermore, it might be possible that the Epstein–Barr virus infection of already primed B-lymphocytes against the NMDA receptor promotes the proliferation of this specific B-cell type and thus increases the synthetization of anti-NMDA receptor antibodies (25).

Another remarkable aspect of our case is the clinical poor response to the broad spectrum of immunosuppressive therapeutics. Although clinical trials are not yet available, clinical experience in the therapy of anti-NMDA receptor encephalitis suggests intravenous methylprednisolone followed by plasma exchange or immunoadsorption therapy and/or intravenous immunoglobulins and an escalation therapy with rituximab and/or cyclophosphamide (26–28). In our case, all the recommended therapies were performed but did not improve the patient's symptoms. White blood cell count showed laboratory efficacy of the immunosuppressive therapy and even anti-NMDAR antibody titers in serum and CSF decreased. However, a correlating positive clinical effect could not be achieved. Even oophorectomy of both sides did not lead to clinical improvement as described in some cases (11, 28).

Early treatment in autoimmune encephalitis patients without previous immunosuppressive therapy has been shown to have a better prognosis (2). Our patient was diagnosed already 3 weeks after the onset of symptoms and had a fatal outcome. In contrast, the other published cases of post-transplant patients with autoimmune encephalitis and full recovery were diagnosed even later (3 and 5 months) after clinical manifestation (5, 7). Thus, the duration of symptoms and onset of a sufficient therapy was rather not the reason for the fatal outcome in our patient.

## Conclusion

Anti-NMDA receptor encephalitis can develop fatally despite previous immunosuppressive therapy. The etiology of anti-NMDA receptor encephalitis is still not fully understood but seems to involve autoimmune mechanisms that are not sufficiently inhibited by mycophenolate mofetil and tacrolimus. In addition, an EBV infection of the cells forming the blood–brain barrier might play a role in the pathogenesis of anti-NMDA receptor encephalitis. An autoimmune encephalitis should be considered in post-transplant patients with neuropsychiatric symptoms.

## Data Availability Statement

The datasets generated for this study are available on request to the corresponding author.

## Ethics Statement

Ethical review and approval was not required for the study on human participants in accordance with the local legislation and institutional requirements. The patients/participants provided their written informed consent to participate in this study.

## Author Contributions

FK participated in the design of the study, collected and analyzed the data, and drafted the manuscript. PS collected the data, analyzed the data, and drafted the manuscript. KJ, MH, FW, and K-WS contributed in drafting the manuscript. MS analyzed the data and contributed in drafting the manuscript. TS conceived the study, analyzed the data, and drafted the manuscript. All authors read and approved the final manuscript.

### Conflict of Interest

The authors declare that the research was conducted in the absence of any commercial or financial relationships that could be construed as a potential conflict of interest.

## Abbreviations

Anti-NMDA receptor: anti-*N*-methyl-D-aspartate receptor
MRI: magnetic resonance imaging
CSF: cerebrospinal fluid
EEG: electroencephalogram
PCR: polymerase chain reaction
EBV: Epstein–Barr virus
CRP: C-reactive protein
OCB: CSF-specific oligoclonal bands
Qalbumin: CSF-serum albumin quotients
PET: positron emission tomography
CT: computed tomography.
